# Survival Analysis of Radiation Therapy in Ovarian Cancer: A SEER Database Analysis

**DOI:** 10.1155/2021/8849039

**Published:** 2021-02-11

**Authors:** Lushi Yu, Hongyun Gong, Qian Li, Honggang Ren, Yi Wang, Haihua He, Tian Li, Qibin Song

**Affiliations:** ^1^Cancer Center, Renmin Hospital of Wuhan University, Wuhan 430060, Hubei, China; ^2^School of Basic Medicine Fourth Military Medical University/Air Force Medical University, Xi'an 710032, Shanxi, China

## Abstract

**Results:**

A total of 20031 ovarian cancer patients were included, with 291 (1.45%) patients who received radiotherapy. The median overall survival (OS) in patients who received radiotherapy was shorter than which in patients without radiotherapy (23 vs. 75 months, *P* < 0.001). The Elderly, nonepithelial pathology, advanced American Joint Committee on Cancer (AJCC) stage, elevated level of CA125, and receiving radiotherapy were risk predictors to survival in both multivariable analyses before and after PSM. Among 11872 patients with III/IV stage, the radiotherapy group also showed a significantly worse prognosis (median OS: 19 vs. 44 months in patients without radiotherapy, *P* < 0.001). Consistent results were observed in stratification analyses on pathology and stage among patients with III/IV stage.

**Conclusions:**

For patients with ovarian cancer, radiotherapy was associated with a poor prognosis regardless of pathology or stage. Considering this is a retrospective study, future studies concerning radiotherapy combination with other new agents in ovarian cancer are needed.

## 1. Introduction

Ovarian cancer (OC), the third most common cause of death in gynecologic cancer, ranked eighth for both cancer incidence and mortality among females in 2018, with 295,414 new cancer cases and 184,799 cancer deaths in 185 countries [1]. The prognoses of OC remain diverse according to pathological type, stage at the first diagnosis, and response to treatment strategy. Epithelial ovarian cancer (EOC) including five major subtypes of high-grade serous, low-grade serous, mucinous, endometrioid, and clear cell ovarian cancer accounts for around 90% of OC. Nonepithelial ovarian cancer mainly consisted of germ cell, sex-cord stromal cancers, and ovarian sarcoma has low morbidity and mortality relative to EOC. Early stage (stage I-II) of OC is highly curable with a 5-year survival rate of 60–80%. Whereas 60–75% of OC firstly diagnosed at stage III/IV, the proportion even up to 80% in serous ovarian cancer. The 5-year survival rate of III/IV stage OC patients is only 19–41% [2, 3].

Debulking surgery and platinum-based chemotherapy are the main treatments for OC, even for recurrent and advanced OC [4–7]. Over the past decade, targeted therapy, such as antiangiogenic agents and poly (ADP-ribose) polymerase-1 (PARP) inhibitors [8, 9], has shown effects in some patients with OC. Immunotherapy is being tried in OC patients, although no significant advantage was observed till now [10]. As a traditional treatment, radiotherapy (RT) is at an inferior place in the treatment of OC due to the dose limit from adjacent normal tissues and is given only for palliative care in most situations. Nowadays, radiation techniques have been improved to enable directed conformal therapy delivery to the local lesion. Thus the position of RT in OC may be reconsidered [11]. However, current studies for RT on OC were limited, and the sample sizes were small [12–14]. Aimed to explore the survival impact of radiotherapy in OC, we analyzed the real-world data from the Surveillance, Epidemiology, and End Results (SEER) Database.

## 2. Materials and Methods

### 2.1. Database and Population Selection

The National Cancer Institute's SEER program collects information about cancer patients in the United States, covering about 34.6% of the population, and is provided as a public service. The SEER^*∗*^Stat software (version 8.3.6) was used to screen eligible patients for analysis. Women who were older than 18 years old with histologically confirmed primary ovarian cancer (ICD-O-3, C569) and had active follow-up record were included preliminarily. Patients who were diagnosed by autopsy or death certificate, had multiple tumors, or without the identified status of radiotherapy were excluded. All included patients were diagnosed from 2010 to 2015.

### 2.2. Variables

Variables involved in the analysis included were age, race, insurance status, marital status, lateral, tumor size, histological subtype, tumor grade, SEER combined summary stage, tumor stage (according to the 6^th^ TNM classification of American Joint Committee on Cancer [AJCC] staging system), treatments, and tumor marker (CA125). Age was categorized into groups of ≤40, 40–60, and >60 according to perimenopausal age. Treatments included surgery, chemotherapy, and radiotherapy. The primary endpoint was overall survival (OS) and the second endpoint was cause specific survival (CSS). The OS was defined as the time interval from first diagnosed as OC to death due to any cause. The CSS was defined as the time interval from diagnosis of OC to OC-related death.

### 2.3. Statistical Analyses

Categorical variables were described as counts (percentage) and compared using the chi-square test or Fisher's exact test. Continuous variables were described as means ± standard deviations (SD) or median (interquartile range) and compared by student's *t*-test or Kruskal–Wallis test. Univariate and multivariate Cox regression analyses were employed to identify independent predictors associated with survival. Survival comparisons were made using Kaplan–Meier analysis and log-rank tests. A 1 : 1 propensity score matching (PSM) analysis was employed to balance baseline variables for further analyses. A two-side *P* value <0.05 was considered statistically significant. Statistical analyses were performed using SPSS 22.0 and figures were made in GraphPad Prism 6.

## 3. Results

### 3.1. Patients and Characteristics

The demographic and clinical characteristics of 20031 included patients were shown in Table 1. The median age was 59 years (IQR 50–68). Most of the patients were white race (80.98%), married (52.26%), insured (94.61%), unilateral (57.96%), and with epithelial pathology (76.68%). Among 20031 patients, 291 (1.45%) patients received RT and 19740 (98.55%) patients without RT. The age, insurance status, and marital status in both RT and non-RT were parallel. Patients with nonepithelial pathology type (52.92% vs. 22.88%, *P* < 0.001), distant stage (67.70% vs. 55.91%, *P* < 0.001), and III/IV stage (50.17% vs. 19.07%, *P* < 0.001) were more likely to receive RT. 99.82% of patients without RT received surgery of primary site, while only 67.70% of patients with RT had operations (*P* < 0.001). Chemotherapy was more common in patients with RT (81.79% vs. 74.66%, *P*=0.006).

Patients and characteristics after PSM were also shown in Table 1. Most variables were balanced, except pathology, SEER combined summary stage, AJCC stage, chemotherapy, and CA125.

### 3.2. Survival Analysis

**Figure 1:**
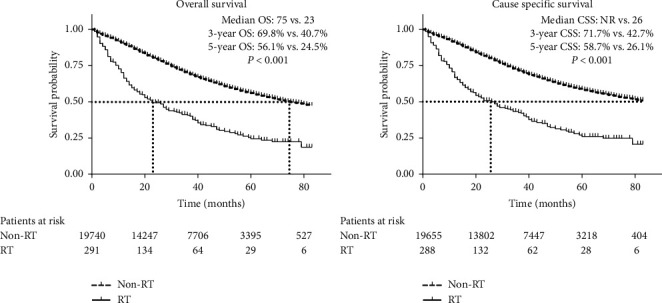
Overall survival and cause specific survival among ovarian cancer patients with or without radiotherapy. RT: radiotherapy; OS: overall survival; CSS: cause specific survival; NR: not reached.

On the whole, the median OS was 75 months in non-RT, while only 23 months in RT (*P* < 0.001), with consistent results in median CSS (not reached vs. 26 months). 1-year, 3-year, and 5-year OS rates in two groups were 90.0% vs. 66.3%, 69.8% vs. 40.7%, and 56.1% vs. 24.5%, respectively (Figure 1).

**Figure 2:**
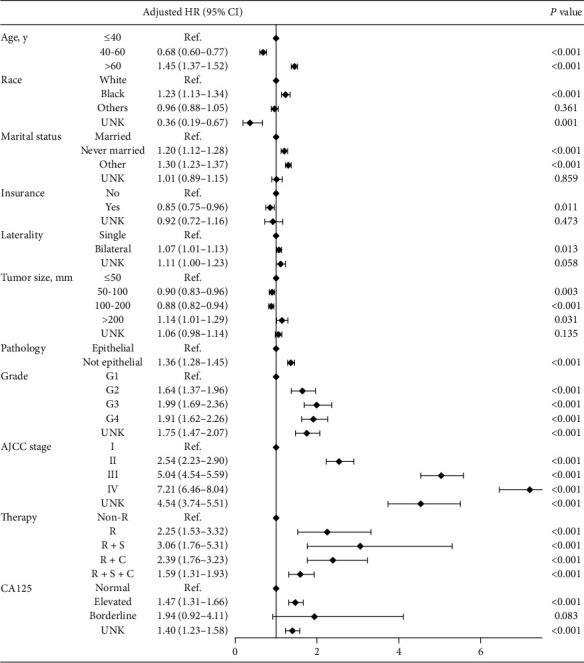
Multivariate survival analysis among ovarian cancer patients. HR: hazard ratio; CI: confidence interval; Ref.: reference; UNK: unknown; R: radiotherapy; S: surgery; C:;chemotherapy.

Univariable (Table 2) and multivariable cox regression were performed to assess risk factors for OS before and after PSM. In the multivariate analysis before PSM, the age, race, marital status, pathology, tumor size, tumor grade, AJCC stage, and level of CA125 were all associated with survival. Besides, no matter RT (R) alone or in combination with surgery (S) or/and chemotherapy (C) impaired survival (R to non-R: HR = 2.25, *P* < 0.001; R + S to non-R: HR = 3.06, *P* < 0.001; R + C to non-R: HR = 2.39, *P* < 0.001; R + S + C to non-R: HR = 1.59, *P* < 0.001) (Figure 2). After PSM, age, insurance, pathology, AJCC stage, and level of CA125 were considered as prognostic factors in multivariate survival analysis. As for therapy, only radiotherapy in combination with surgery showed significantly unfavorable effects on overall survival (Supplementary Figure 1).

### 3.3. Radiotherapy in III/IV Stage Ovarian Cancer

**Figure 3:**
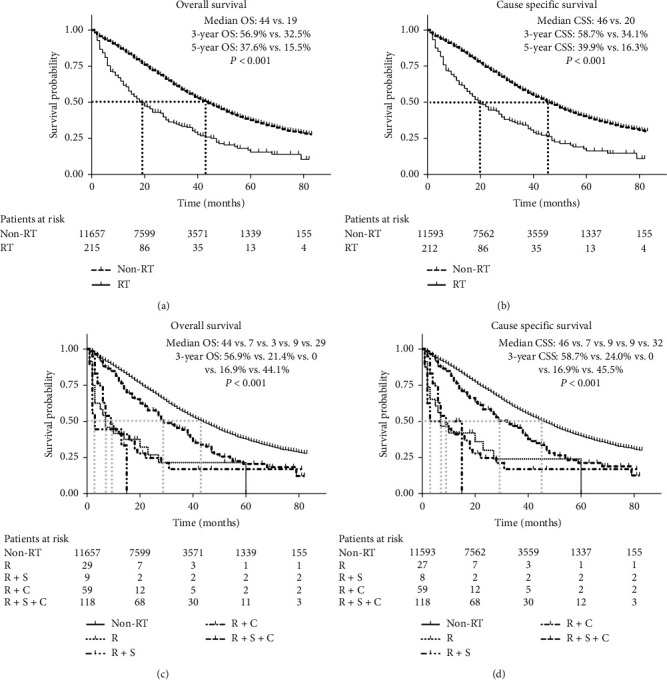
Overall survival and cause specific survival for radiotherapy among ovarian cancer patients with III/IV stage. OS and CSS for RT and non-RT among ovarian cancer patients with III/IV stage are shown in (a) and (b), respectively; patients with RT were further divided into four groups according the therapies combined with RT, and the OS and CSS are shown in (c) and (d). RT: radiotherapy; R: radiotherapy; S: surgery; C: chemotherapy; OS: overall survival; CSS: cause specific survival.

According to baseline characteristics of patients, radiotherapy was common in III/IV stage ovarian cancer. Therefore, we further analyzed OC patients with III/IV stage. Among 11872 patients with III/IV OC, 215 patients received radiotherapy (Supplementary Table 1). Significant statistical differences were in both OS (median OS 44 vs. 19 months; 3-year OS 56.9% vs. 32.5%; 5-year OS 37.6% vs. 15.5%; *P* < 0.001) and CSS (median CSS 46 vs. 20 months; 3-year CSS 58.7% vs. 34.1%; 5-year CSS 39.9% vs. 16.3%; *P* < 0.001) between non-RT and RT groups in general (Figures 3(a) and 3(b)). When differentiating RT monotherapy and combination therapy, RT combination with both surgery and chemotherapy can obtain a relatively good survival (median OS 29 months, median CSS 32 months) compared with RT monotherapy and RT combination with surgery or chemotherapy, though still worse than non-RT (Figures 3(c) and 3(d).

**Figure 4:**
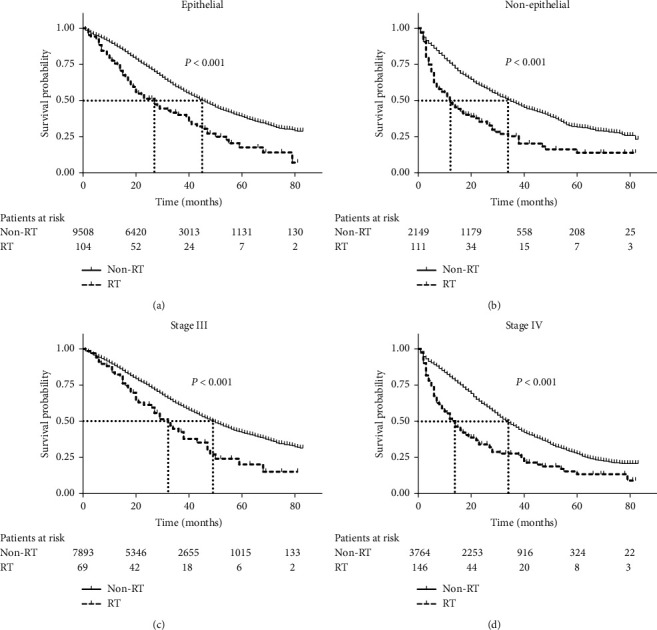
Stratified survival analyses by radiotherapy among ovarian cancer patients with III/IV stage. (a). (b). Kaplan–Meier curves for overall survival in stratification analysis according to pathological type. (c). (d). Kaplan–Meier curves for overall survival in stratification analysis according to stage. RT: radiotherapy.

Stratification analyses were carried out to control confounders. In stratification analysis according to pathological type, 9612 (80.96%) patients were EOC, with the median OS of 45 and 27 months (*P* < 0.001) in non-RT and RT groups, respectively (Figure 4(a)). The median OS of non-EOC was only 35 and 12 months (*P* < 0.001) in two groups, respectively (Figure 4(b)). In stratification analysis according to AJCC stage, 7962 (67.07%) patients were first diagnosed as III stage. The median OS of the III stage was 50 and 32 months (*P* < 0.001) in non-RT and RT groups, respectively (Figure 4(c)). For 3910 (32.93%) patients with stage IV, the median OS was reduced to 34 and 14 months (*P* < 0.001) in two groups, respectively (Figure 4(d)). The CSS in the stratification analysis was similar to OS (Supplementary Figure 2).

## 4. Discussion

Historically, conventional whole abdominal radiotherapy (WART) was used in ovarian cancer to reduce recurrence [15], while it has been proved to bring several severe acute and late toxic effects [16–18] and has almost been abandoned nowadays. According to National Comprehensive Cancer Network (NCCN) clinical practice guidelines for ovarian cancer, radiation therapy is only recommended for limited disease in malignant sex-cord stromal tumors and is considered as a palliative localized strategy for patients with recurrent disease.

Given the advance of techniques of radiation therapy, researches on radiation therapy for OC are still ongoing. The OVAR-IMRT-01 study, a phase I study, evaluated the clinical feasibility and acceptable toxicity of WART using intensity modulated radiotherapy (IMRT) as consolidation therapy in advanced OC [12]. Following that, the multicenter, single-arm, phase II study, OVAR-IMRT-02 showed that more than 70% of 20 included patients could tolerate intensity modulated WART and a mean decrease of global health status score was 18.1 points [13]. Chang made a prospective phase II trial on involved-field radiotherapy (IFRT) in 30 patients with recurrent EOC. The results showed an overall response rate (ORR) of 85.7%, median progress-free survival (PFS) of 7 months, and a 3-year OS rate of 55.8%, revealing the effectiveness of IFRT for local control in EOC [19]. Kim retrospectively analyzed 61 recurrent EOC patients who were treated with IFRT. They found that the 2-year in-field control, PFS, and OS rates were 42.7%, 24.2%, and 78.9%, respectively. The elevated CA125 level was considered to be related to a worse OS [20]. Chundury gave IMRT to 33 recurrent chemorefractory OC, finding that 2-year local control and OS rates were 82% and 63%, respectively, with limited radiation related toxicity [21]. Although positive results on RT are shown in the researches above, the sample size was small and all of those were single-arm studies without control groups. Therefore, phase III randomized controlled clinical trials and big sample size real-world studies are needed to provide more powerful evidence.

In this real-world research that included 20031 OC patients, we found that the median OS of the RT group was 23 months, and 1-year, 2-year, and 3-year OS rates were 66.3%, 49.4%, and 40.7%, respectively, which were slightly worse than reported previously [14, 19]. The difference could be explained by different sample sizes, radiation fields, dose and modality, and so on. Non-RT as a control group in this study had better survival, with a median OS of 75 months and a 5-year OS rate of 56.1%. On the whole, radiation therapy was commonly given to ovarian cancer patients with nonepithelial type, distant stage and AJCC III/IV stage, so at first, we blamed the poor survival to worse pathological type and more advanced stage in RT group. However, after subgroup and stratification analysis, similar results were obtained. For III/IV OC, median OS was 44 versus 19 months and 3-year OS rates were 56.9% versus 32.5% in non-RT and RT, respectively. For EOC, median OS was 45 versus 27 months in non-RT and RT group, respectively.

Multivariable survival analysis also supported the result above. Except for old age, poor pathology, advanced AJCC stage, and high level of CA125, radiotherapy also was a risk factor for poor survival. Especially, RT combination with surgery may result in shorter survival in III/IV OC patients, though the number of patients in the analysis was only 9 in this study. Thus, we thought that radiotherapy could not bring survival benefits for ovarian cancer compared to patients without radiotherapy.

Radiotherapy, as well as its combination with surgery or chemotherapy, in this study, did not bring survival benefits to ovarian cancer patients, but some preclinical studies showed that PARP inhibitors might be sensitizing agents for radiotherapy; others also agreed that radiotherapy could promote tumor immunity cycle in OC, which implied the potential synergy of radiotherapy with targeted therapy or immunotherapy in OC [22–24].

Our study with a big sample size and real-world data provided some information for clinical practice on radiotherapy in OC. However, there were still several limitations. Firstly, the proportion of ovarian cancer patients who received RT in this study was small, only 1.45% (291/20031). Thus some underlying confounding factors could not be ignored. The types of surgery in this study varied from local tumor destruction to cytoreductive surgery, and regimens of chemotherapy were unknowable. Both chemotherapy and surgery can influence survival, especially debulking surgery, which is acknowledged as improving the survival of ovarian cancer. Although we made PSM analysis to balance baseline variables as many as possible, the rates of surgery and chemotherapy were not balanced at the same time. So, surgery and chemotherapy were likely to be confounding factors to impaired survival in RT. Also, the difference in the surgeons who conducted the surgery (gynecological oncologist, general obstetrics and gynecology physician or general surgeon) could affect the scope of surgery, the status of residual lesions, and even survival. Secondly, we did not explore the effect of sequence between radiotherapy and surgery on survival because only a small number of patients received surgery after or during radiotherapy. Due to the lack of detail information of field, dose and modality of radiotherapy, and the information of adverse events, we were not able to compare the effect of different radiation patterns on survival and evaluated the safety of RT in OC. All of those might influence the effect of RT on OC. Thirdly, some patients' death causes were unknown which may affect the reliability of CSS, but the proportions were small (0.43% [88/20031]). Thus the results of CSS in this study could also be considered as references. Lastly, this study remained a retrospective study, and potential biases were unavoidable. Large randomized controlled clinical trials are looked forward to.

## 5. Conclusions

Radiotherapy was often given to ovarian cancer patients with nonepithelial pathology and advanced stage, while it was associated with poor prognosis compared to patients without radiotherapy. The impact of radiotherapy in combination with other new agents in ovarian cancer is exploring. Large randomized controlled clinical trials are needed.

## Figures and Tables

**Table 1:** Baseline characteristics of patients with ovarian cancer before and after propensity score matching analysis.

	Total (*N* = 20031)	Before PSM			After PSM		
		Non-RT (*N* = 19740)	RT (*N* = 291)	*P* value	Non-RT (*N* = 218)	RT (*N* = 218)	*P* value
Age, y, median (IQR)	59 (50–68)	59 (50–68)	59 (50–70)	0.387	58 (49–68)	58 (49–68)	0.786
≤40	2056 (10.26%)	2030 (10.28%)	26 (8.93%)	0.640	102 (46.79%)	107 (49.08%)	0.784
40–60	8903 (44.45%)	8767 (44.41%)	136 (46.74%)	—	24 (11.01%)	20 (9.17%)	—
>60	9072 (45.29%)	8943 (45.30%)	129 (44.33%)	—	92 (42.20%)	91 (41.74%)	—

Race				0.031			0.096
White	16221 (80.98%)	16001 (81.06%)	220 (75.60%)	—	162 (74.31%)	169 (77.52%)	—
Black	1589 (7.93%)	1553 (7.87%)	36 (12.37%)	—	13 (5.96%)	20 (9.17%)	—
Others	2094 (10.45%)	2060 (10.44%)	34 (11.68%)	—	43 (19.72%)	28 (12.84%)	—
UNK	127 (0.63%)	126 (0.64%)	1 (0.34%)	—	0	1 (0.46%)	—

Marital status				0.545			0.927
Married	10468 (52.26%)	10323 (52.29%)	145 (49.83%)	—	112 (51.38%)	115 (52.75%)	—
Never married	4207 (21.00%)	4141 (20.98%)	66 (22.68%)	—	46 (21.10%)	49 (22.48%)	—
Other	4430 (22.12%)	4360 (22.09%)	70 (24.05%)	—	54 (24.77%)	48 (22.02%)	—
UNK	926 (4.62%)	916 (4.64%)	10 (3.44%)	—	6 (2.75%)	6 (2.75%)	—

Insurance				0.853			0.182
No	761 (3.80%)	750 (3.80%)	11 (3.78%)	—	2 (0.92%)	7 (3.21%)	—
Yes	18951 (94.61%)	18677 (94.61%)	274 (94.16%)	—	214 (98.17%)	208 (95.41%)	—
UNK	319 (1.59%)	313 (1.59%)	6 (2.06%)	—	2 (0.92%)	3 (1.38%)	—

Laterality				<0.001			0.077
Unilateral	11610 (57.96%)	11468 (58.10%)	142 (48.80%)	—	121 (55.50%)	125 (57.34%)	—
Bilateral	7504 (37.46%)	7426 (37.62%)	78 (26.80%)	—	82 (37.61%)	66 (30.28%)	—
UNK	917 (4.58%)	846 (4.29%)	71 (24.40%)	—	15 (6.88%)	27 (12.39%)	—

Tumor size, mm				<0.001			0.156
≤50	3881 (19.37%)	3838 (19.44%)	43 (14.78%)	—	42 (19.27%)	33 (15.14%)	—
50–100	4770 (23.81%)	4711 (23.87%)	59 (20.27%)	—	54 (24.77%)	54 (24.77%)	—
100–200	5867 (29.29%)	5792 (29.34%)	75 (25.77%)	—	48 (22.02%)	63 (28.90%)	—
>200	1263 (6.31%)	1246 (6.31%)	17 (5.84%)	—	8 (3.67%)	15 (6.88%)	—
UNK	4250 (21.22%)	4153 (21.04%)	97 (33.33%)	—	66 (30.28%)	53 (24.31%)	—

Pathology				<0.001			<0.001
Epithelial	15360 (76.68%)	15223 (77.12%)	137 (47.08%)	—	156 (71.56%)	119 (54.59%)	—
Not epithelial	4671 (23.32%)	4517 (22.88%)	154 (52.92%)	—	62 (28.44%)	99 (45.41%)	—

Grade				<0.001			0.192
G1	1660 (8.29%)	1653 (8.37%)	7 (2.41%)	—	15 (6.88%)	6 (2.75%)	—
G2	2423 (12.10%)	2408 (12.20%)	15 (5.15%)	—	21 (9.63%)	14 (6.42%)	—
G3	6429 (32.10%)	6322 (32.03%)	107 (36.77%)	—	82 (37.61%)	92 (42.20%)	—
G4	4885 (24.39%)	4833 (24.48%)	52 (17.87%)	—	43 (19.72%)	48 (22.02%)	—
UNK	4634 (23.13%)	4524 (22.92%)	110 (37.80%)	—	57 (26.15%)	58 (26.61%)	—

SEER combined summary stage				<0.001			<0.001	
Local	3708 (18.51%)	3700 (18.74%)	8 (2.75%)	—	37 (16.97%)	8 (3.67%)	—
Regional	4874 (24.33%)	4793 (24.28%)	81 (27.84%)	—	50 (22.94%)	73 (33.49%)	—
Distant	11234 (56.08%)	11037 (55.91%)	197 (67.70%)	—	128 (58.72%)	133 (61.01%)	—
UNK	215 (1.07%)	210 (1.06%)	5 (1.72%)	—	3 (1.38%)	4 (1.83%)	—

AJCC stage				<0.001			<0.001
I	5827 (29.09%)	5806 (29.41%)	21 (7.22%)	—	60 (27.52%)	21 (9.63%)	—
II	1956 (9.76%)	1915 (9.70%)	41 (14.09%)	—	15 (6.88%)	40 (18.35%)	—
III	7962 (39.75%)	7893 (39.98%)	69 (23.71%)	—	77 (35.32%)	61 (27.98%)	—
IV	3910 (19.52%)	3764 (19.07%)	146 (50.17%)	—	59 (27.06%)	86 (39.45%)	—
UNK	376 (1.88%)	362 (1.83%)	14 (4.81%)	—	7 (3.21%)	10 (4.59%)	—

T				<0.001			<0.001
T0	29 (0.14%)	20 (0.10%)	9 (3.09%)	—	1 (0.46%)	1 (0.46%)	—
T1	6261 (31.26%)	6225 (31.53%)	36 (12.37%)	—	73 (33.49%)	34 (15.60%)	—
T2	2745 (13.70%)	2663 (13.49%)	82 (28.18%)	—	26 (11.93%)	66 (30.28%)	—
T3	10500 (52.42%)	10380 (52.58%)	120 (41.24%)	—	102 (46.79%)	97 (44.50%)	—
Tx/NA	496 (2.48%)	452 (2.29%)	44 (15.12%)	—	16 (7.34%)	20 (9.17%)	—

N				<0.001			0.343
N0	14628 (73.03%)	14477 (73.34%)	151 (51.89%)	—	137 (62.84%)	125 (57.34%)	—
N1	4273 (21.33%)	4178 (21.17%)	95 (32.65%)	—	58 (26.61%)	72 (33.03%)	—
Nx/NA	1130 (5.64%)	1085 (5.50%)	45 (15.46%)	—	23 (10.55%)	21 (9.63%)	—

M				<0.001			0.022
M0	16062 (80.19%)	15920 (80.65%)	142 (48.80%)	—	155 (71.10%)	129 (59.17%)	—
M1	3910 (19.52%)	3764 (19.07%)	146 (50.17%)	—	59 (27.06%)	86 (39.45%)	—
NA	59 (0.29%)	56 (0.28%)	3 (1.03%)	—	4 (1.83%)	3 (1.38%)	—

Metastasis							
Bone	99 (0.49%)	61 (0.31%)	38 (13.06%)	<0.001	1 (0.46%)	18 (8.26%)	<0.001
Brain	33 (0.16%)	7 (0.04%)	26 (8.93%)	<0.001	0	13 (5.96%)	<0.001
Liver	893 (4.46%)	854 (4.33%)	39 (13.40%)	<0.001	13 (5.96%)	20 (9.17%)	0.139
Lung	704 (3.51%)	666 (3.37%)	38 (13.06%)	<0.001	17 (7.80%)	19 (8.72%)	0.877

Surgery				<0.001			1.000
Yes	19902 (99.36%)	19705 (99.82%)	197 (67.70%)	—	197 (90.37%)	197 (90.37%)	—
No	129 (0.64%)	35 (0.18%)	94 (32.30%)	—	21 (9.63%)	21 (9.63%)	—

Chemotherapy				0.006			0.014
Yes	14976 (74.76%)	14738 (74.66%)	238 (81.79%)	—	160 (73.39%)	182 (83.49%)	—
No/UNK	5055 (25.24%)	5002 (25.34%)	53 (18.21%)	—	58 (26.61%)	36 (16.51%)	—

CA125, U/mL				0.085			0.001
Normal	2117 (10.57%)	2089 (10.58%)	28 (9.62%)	—	38 (17.43%)	24 (11.01%)	—
Elevated	13444 (67.12%)	13264 (67.19%)	180 (61.86%)	—	146 (66.97%)	130 (59.63%)	—
Borderline	28 (0.14%)	28 (0.14%)	0	—	0	0	—
UNK	4442 (22.18%)	4359 (22.08%)	83 (28.52%)	—	34 (15.60%)	64 (29.36%)	—

Abbreviations: PSM: propensity score matching; RT: radiotherapy; IQR: interquartile range; UNK: unknown; NA: not applicable.

**Table 2:** Univariable Cox regression analysis for patients with ovarian cancer before and after propensity score matching analysis.

		Before PSM		After PSM	
		Unadjusted HR (95% CI)	*P* value	Unadjusted HR (95% CI)	*P* value
Age, y	≤40	Ref.		Ref.	
	40–60	0.46 (0.41–0.52)	<0.001	0.58 (0.33–1.01)	0.056
	>60	1.78 (1.69–1.87)	<0.001	1.88 (1.45–2.42)	<0.001

Race	White	Ref.		Ref.	
	Black	1.26 (1.16–1.36)	<0.001	1.22 (0.79–1.88)	0.364
	Others	0.79 (0.72–0.86)	<0.001	0.64 (0.44–0.93)	0.021
	UNK	0.25 (0.13–0.46)	<0.001	0 (0-NR)	0.946

Marital status	Married	Ref.		Ref.	
	Never married	0.97 (0.91–1.04)	0.405	0.92 (0.66–1.27)	0.600
	Other	1.53 (1.45–1.62)	<0.001	1.37 (1.02–1.84)	0.035
	UNK	0.99 (0.88–1.12)	0.874	1.14 (0.50–2.60)	0.746

Insurance	No	Ref.		Ref.	
	Yes	1.04 (0.92–1.18)	0.566	0.29 (0.13–0.61)	0.001
	UNK	1.07 (0.85–1.34)	0.583	0.44 (0.11–1.71)	0.238

Laterality	Single	Ref.		Ref.	
	Bilateral	1.94 (1.84–2.03)	<0.001	1.45 (1.11–1.89)	0.007
	UNK	2.84 (2.58–3.12)	<0.001	4.10 (2.78–6.04)	<0.001

Tumor size, mm	≤50	Ref.		Ref.	
	50–100	0.93 (0.86–1.00)	0.040	0.96 (0.64–1.46)	0.862
	100–200	0.75 (0.70–0.81)	<0.001	1.30 (0.87–1.95)	0.193
	>200	0.72 (0.64–0.81)	<0.001	1.43 (0.76–2.68)	0.267
	UNK	1.30 (1.21–1.39)	<0.001	1.58 (1.07–2.33)	0.021

Pathology	Epithelial	Ref.		Ref.	
	Not epithelial	1.10 (1.04–1.17)	<0.001	1.62 (1.26–2.08)	<0.001

Grade	G1	Ref.		Ref.	
	G2	2.54 (2.12–3.04)	<0.001	1.24 (0.42–3.62)	0.697
	G3	5.53 (4.70–6.50)	<0.001	3.53 (1.44–8.66)	0.006
	G4	5.42 (4.60–6.38)	<0.001	3.62 (1.45–9.06)	0.006
	UNK	4.33 (3.67–5.11)	<0.001	3.99 (1.61–9.90)	0.003

SEER combined summary stage	Local	Ref.		Ref.	
	Regional	2.67 (2.33–3.05)	<0.001	4.59 (1.82–11.60)	0.001
	Distant	9.07 (8.04–10.23)	<0.001	14.35 (5.89–34.97)	<0.001
	UNK	5.91 (4.59–7.62)	<0.001	9.40 (2.52–35.04)	0.001

AJCC stage	I	Ref.		Ref.	
	II	3.10 (2.73–3.53)	<0.001	6.64 (3.11–14.19)	<0.001
	III	6.85 (6.23–7.53)	<0.001	9.69 (4.87–19.26)	<0.001
	IV	10.71 (9.71–11.82)	<0.001	16.62 (8.39–32.92)	<0.001
	UNK	6.26 (5.21–7.53)	<0.001	11.00 (4.55–26.59)	<0.001

T	T0	Ref.		Ref.	
	T1	0.16 (0.09–0.30)	<0.001	0.20 (0.03–1.48)	0.114
	T2	0.57 (0.32–1.04)	0.065	0.67 (0.09–4.88)	0.693
	T3	1.11 (0.61–2.01)	0.730	1.19 (0.17–8.49)	0.865
	Tx/NA	1.11 (0.61–2.03)	0.737	1.72 (0.23–12.67)	0.595

N	N0	Ref.		Ref.	
	N1	1.83 (1.73–1.93)	<0.001	1.65 (1.25–2.16)	<0.001
	Nx/NA	2.73 (2.51–2.96)	<0.001	2.71 (1.86–3.95)	<0.001

M	M0	Ref.		Ref.	
	M1	2.69 (2.56–2.83)	<0.001	2.82 (2.19–3.64)	<0.001
	NA	1.95 (1.32–2.89)	0.001	2.51 (1.03–6.15)	0.043

Surgery	No	Ref.		Ref.	
	Yes	0.14 (0.11–0.16)	<0.001	0.16 (0.11–0.23)	<0.001

Radiation	No	Ref.		Ref.	
	Yes	2.70 (2.34–3.12)	<0.001	1.72 (1.33–2.22)	<0.001

Chemotherapy	Yes	Ref.		Ref.	
	No/UNK	0.76 (0.72–0.81)	<0.001	1.21 (0.90–1.64)	0.208

Therapy	Non-R	Ref.		Ref.	
	R	6.83 (4.68–9.97)	<0.001	4.90 (2.75–8.74)	<0.001
	R + S	2.77 (1.61–4.77)	<0.001	2.10 (1.18–3.74)	0.012
	R + C	5.50 (4.10–7.38)	<0.001	4.06 (1.48–11.12)	0.007
	S + C + R	1.97 (1.62–2.38)	<0.001	1.53 (1.17–2.01)	0.002

CA125, U/mL	Normal	Ref.		Ref.	
	Elevated	3.23 (2.88–3.62)	<0.001	3.77 (2.22–6.38)	<0.001
	Borderline	1.66 (0.78–3.51)	0.186	—	—
	UNK	2.28 (2.02–2.59)	<0.001	3.67 (2.08–6.47)	<0.001

Abbreviations: PSM: propensity score matching; HR: hazard ratio; CI: confidence interval; Ref.: reference; UNK: unknown; NA: not applicable; R: radiotherapy; S: surgery; C: chemotherapy.

## Data Availability

The data used and/or analyzed in this study are available in the Surveillance, Epidemiology, and End Results (SEER) Database of the National Cancer Institute (http://seer.cancer.gov).

## Supplementary Materials

Supplementary Materials Supplementary Table 1: Baseline characteristics of ovarian cancer patients with III/IV stage. Supplementary Figure 1: Multivariate survival analysis among ovarian cancer patients with III/IV stage. Supplementary Figure 2: Stratified cause specific survival analyses by radiotherapy among ovarian cancer patients with III/IV stage. A.B. Kaplan–Meier curves for cause specific survival in stratification analysis according to pathological type. C.D. Kaplan–Meier curves for cause specific survival in stratification analysis according to stage.
