# Supplementary material for: Survival Analysis of Radiation Therapy in Ovarian Cancer: A SEER Database Analysis
Source: J Oncol. 2021 Feb 11;2021:8849039. doi: 10.1155/2021/8849039 (PMC7892241; doi:10.1155/2021/8849039)
Supplement: Supplementary Materials — Supplementary Table 1: Baseline characteristics of ovarian cancer patients with III/IV stage. Supplementary Figure 1: Multivariate survival analysis among ovarian cancer patients with III/IV stage. Supplementary Figure 2: Stratified cause specific survival analyses by radiotherapy among ovarian cancer patients with III/IV stage. A.B. Kaplan–Meier curves for cause specific survival in stratification analysis according to pathological type. C.D. Kaplan–Meier curves for cause specific survival in stratification analysis according to stage. [file 8849039.f1.docx]

**Supplementary Material**

**Suppl. Table 1.** Baseline characteristics of ovarian cancer patients with III/IV stage.

|  | Total  N=11872 | Non-RT  N=11657 | RT  N=215 | *P* value |
| --- | --- | --- | --- | --- |
| Age, y, median (IQR) | 62 (53-70) | 62 (53-70) | 59 (51-70) | 0.247 |
| ≤40 | 4890 (41.19%) | 4789 (41.08%) | 101 (46.98%) | 0.050 |
| 40-60 | 643 (5.42%) | 627 (5.38%) | 16 (7.44%) | - |
| >60 | 6339 (53.39%) | 6241 (53.54%) | 98 (45.58%) | - |
| Race |  |  |  | 0.009 |
| White | 9807 (82.61%) | 9645 (82.74%) | 162 (75.35%) | - |
| Black | 962 (8.10%) | 931 (7.99%) | 31 (14.42%) | - |
| Others | 1064 (8.96%) | 1042 (8.94%) | 22 (10.23%) | - |
| UNK | 39 (0.33%) | 39 (0.33%) | 0 | - |
| Marital status |  |  |  | 0.180 |
| Married | 6324 (53.27%) | 6219 (53.35%) | 105 (48.84%) | - |
| Never married | 2164 (18.23%) | 2113 (18.13%) | 51 (23.72%) | - |
| Other | 2891 (24.35%) | 2839 (24.35%) | 52 (24.19%) | - |
| UNK | 493 (4.15%) | 486 (4.17%) | 7 (3.26%) | - |
| Insurance |  |  |  | 0.389 |
| No | 394 (3.32%) | 384 (3.29%) | 10 (4.65%) | - |
| Yes | 11330 (95.43%) | 11129 (95.47%) | 201 (93.49%) | - |
| UNK | 148 (1.25%) | 144 (1.24%) | 4 (1.86%) | - |
| Laterality |  |  |  | <0.001 |
| Single | 4670 (39.34%) | 4584 (39.32%) | 86 (40.00%) | - |
| Bilateral | 6449 (54.32%) | 6382 (54.75%) | 67 (31.16%) | - |
| UNK | 753 (6.34%) | 691 (5.93%) | 62 (28.84%) | - |
| Tumor size, mm |  |  |  |  |
| ≤50 | 2449 (20.63%) | 2412 (20.69%) | 37 (17.21%) | 0.007 |
| 50-100 | 3027 (25.50%) | 2983 (25.59%) | 44 (20.47%) | - |
| 100-200 | 2946 (24.81%) | 2899 (24.87%) | 47 (21.86%) | - |
| >200 | 460 (3.87%) | 450 (3.86%) | 10 (4.65%) | - |
| UNK | 2990 (25.19%) | 2913 (24.99%) | 77 (35.81%) | - |
| Pathology |  |  |  | <0.001 |
| Epithelial | 9612 (80.96%) | 9508 (81.56%) | 104 (48.37%) | - |
| Not epithelial | 2260 (19.04%) | 2149 (18.44%) | 111 (51.63%) | - |
| Grade |  |  |  | <0.001 |
| G1 | 268 (2.26%) | 264 (2.26%) | 4 (1.86%) | - |
| G2 | 853 (7.18%) | 846 (7.26%) | 7 (3.26%) | - |
| G3 | 4543 (38.27%) | 4461 (38.27%) | 82 (38.14%) | - |
| G4 | 3693 (31.11%) | 3654 (31.35%) | 39 (18.14%) | - |
| UNK | 2515 (21.18%) | 2432 (20.86%) | 83 (38.60%) | - |
| SEER Combined Summary Stage | |  |  | 0.037 |
| Regional | 661 (5.57%) | 642 (5.51%) | 19 (8.84%) | - |
| Distant | 11211 (94.43%) | 11015 (94.49%) | 196 (91.16%) | - |
| AJCC Stage |  |  |  | <0.001 |
| III | 7962 (67.07%) | 7893 (67.71%) | 69 (32.09%) | - |
| IV | 3910 (32.93%) | 3764 (32.29%) | 146 (67.91%) | - |
| T |  |  |  | <0.001 |
| T0 | 29 (0.24%) | 20 (0.17%) | 9 (4.19%) | - |
| T1 | 384 (3.23%) | 370 (3.17%) | 14 (6.51%) | - |
| T2 | 734 (6.18%) | 698 (5.99%) | 36 (16.74%) | - |
| T3 | 10500 (88.44%) | 10380 (89.05%) | 120 (55.81%) | - |
| Tx/NA | 225 (1.90%) | 189 (1.62%) | 36 (16.74%) | - |
| N |  |  |  | <0.001 |
| N0 | 6785 (57.15%) | 6698 (57.46%) | 87 (40.47%) | - |
| N1 | 4273 (35.99%) | 4178 (35.84%) | 95 (44.19%) | - |
| Nx/NA | 814 (6.86%) | 781 (6.70%) | 33 (15.35%) | - |
| M |  |  |  | <0.001 |
| M0 | 7962 (67.07%) | 7893 (67.71%) | 69 (32.09%) | - |
| M1 | 3910 (32.93%) | 3764 (32.29%) | 146 (67.91%) | - |
| Metastasis |  |  |  |  |
| Bone | 99 (0.83%) | 61 (0.52%) | 38 (17.67%) | <0.001 |
| Brain | 33 (0.28%) | 7 (0.06%) | 26 (12.09%) | <0.001 |
| Liver | 892 (7.51%) | 853 (7.32%) | 39 (18.14%) | <0.001 |
| Lung | 700 (5.90%) | 662 (5.68%) | 38 (17.67%) | <0.001 |
| Surgery |  |  |  | <0.001 |
| Yes | 11751 (98.98%) | 11624 (99.72%) | 127 (59.07%) | - |
| No | 121 (1.02%) | 33 (0.28%) | 88 (40.93%) | - |
| Chemotherapy |  |  |  |  |
| Yes | 10322 (86.94%) | 10145 (87.03%) | 177 (82.33%) | 0.051 |
| No/UNK | 1550 (13.06%) | 1512 (12.97%) | 38 (17.67%) | - |
| CA125 |  |  |  | 0.085 |
| Normal | 455 (3.83%) | 441 (3.78%) | 14 (6.51%) | - |
| Elevated | 9377 (78.98%) | 9234 (79.21%) | 143 (66.51%) | - |
| Borderline | 8 (0.07%) | 8 (0.07%) | 0 | - |
| UNK | 2032 (17.12%) | 1974 (16.93%) | 58 (26.98%) | - |

Abbreviations: RT, radiotherapy; IQR, interquartile range; UNK, unknown; NA, not applicable.

**

Suppl. Figure 1.** Multivariate survival analysis among ovarian cancer patients with III/IV stage.

HR, hazard ratio; CI, confidence interval; Ref., reference; UNK, unknown; R, radiotherapy; S, surgery; C, chemotherapy

**

Suppl. Figure 2.** Stratified cause specific survival analyses by radiotherapy among ovarian cancer patients with III/IV stage.

A.B. Kaplan-Meier curves for cause specific survival in stratification analysis according to pathological type. C.D. Kaplan-Meier curves for cause specific survival in stratification analysis according to stage.

RT, radiotherapy.
